# Atomic layer deposition of metal oxides for efficient perovskite single-junction and perovskite/silicon tandem solar cells†

**DOI:** 10.1039/d0ra00939c

**Published:** 2020-04-14

**Authors:** Mohammad I. Hossain, Adnan Mohammad, Wayesh Qarony, Saidjafarzoda Ilhom, Deepa R. Shukla, Dietmar Knipp, Necmi Biyikli, Yuen Hong Tsang

**Affiliations:** Department of Applied Physics, The Hong Kong Polytechnic University Hung Hom Kowloon Hong Kong yuen.tsang@polyu.edu.hk; Department of Electrical and Computer Engineering, University of Connecticut Storrs CT 06269 USA necmi.biyikli@uconn.edu; Department of Materials Science and Engineering, University of Connecticut Storrs CT 06269 USA; Geballe Laboratory for Advanced Materials, Department of Materials Science and Engineering, Stanford University Stanford CA 94305 USA dknipp@stanford.edu

## Abstract

Aluminum-doped and undoped zinc oxide films were investigated as potential front and rear contacts of perovskite single and perovskite/silicon tandem solar cells. The films were prepared by atomic layer deposition (ALD) at low (<200 °C) substrate temperatures. The deposited films were crystalline with a single-phase wurtzite structure and exhibit excellent uniformity and low surface roughness which was confirmed by XRD and SEM measurements. Necessary material characterizations allow for realizing high-quality films with low resistivity and high optical transparency at the standard growth rate. Spectroscopic ellipsometry measurements were carried out to extract the complex refractive index of the deposited films, which were used to study the optics of perovskite single junction and perovskite/silicon tandem solar cells. The optics was investigated by three-dimensional finite-difference time-domain simulations. Guidelines are provided on how to realize perovskite solar cells exhibiting high short-circuit current densities. Furthermore, detailed guidelines are given for realizing perovskite/silicon tandem solar cells with short-circuit current densities exceeding 20 mA cm^−2^ and potential energy conversion efficiencies beyond 31%.

## Abstract 2

The necessity of thin and highly doped metal oxide films is discussed for realizing efficient perovskite single and perovskite/silicon tandem solar cells.

## Graphical abstract

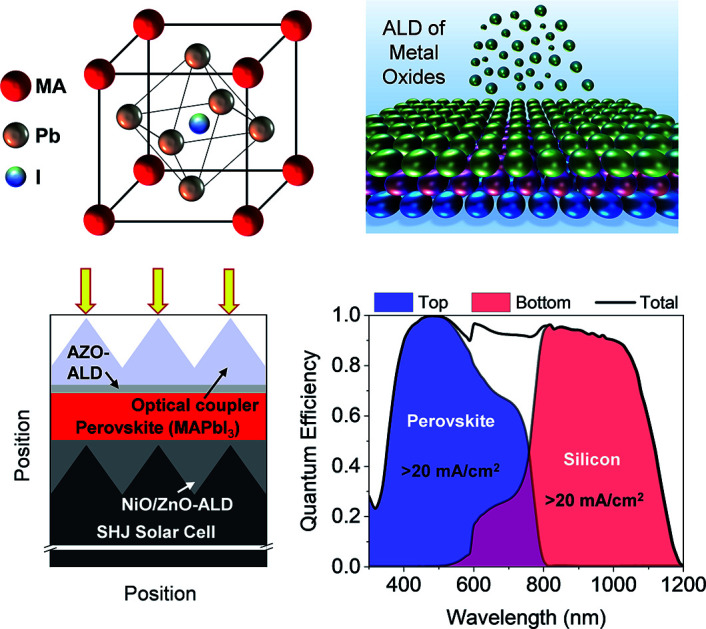

## 1. Introduction

Organometallic halide perovskites have been projected as one of the most promising material systems for future solar cells, and exhibit excellent optoelectronic properties like a large diffusion length (>1 μm) and a small penetration depth (<1 μm).^1–3^ Record perovskite solar cells (PSCs) with energy conversion efficiency (ECE) exceeding 24% have been demonstrated.^4–6^ Furthermore, the tunable bandgap of the perovskite material system allows for the realization of perovskite/silicon and perovskite/perovskite tandem solar cells (TSCs).^7–10^ In particular, the combination of PSCs with crystalline silicon (c-Si) solar cells to form perovskite/silicon TSC is a promising route to achieve high ECEs while the manufacturing cost remains low.^7,8,11^ Up to now, perovskite/silicon TSCs with ECEs of 23.6% and 25.2% have been demonstrated.^7,8^ According to the Shockley–Queisser (S–Q) theory, the ECE of perovskite/silicon TSCs can reach beyond 35% if an optimum bandgap of perovskite is selected.^12,13^

**Fig. 1:**
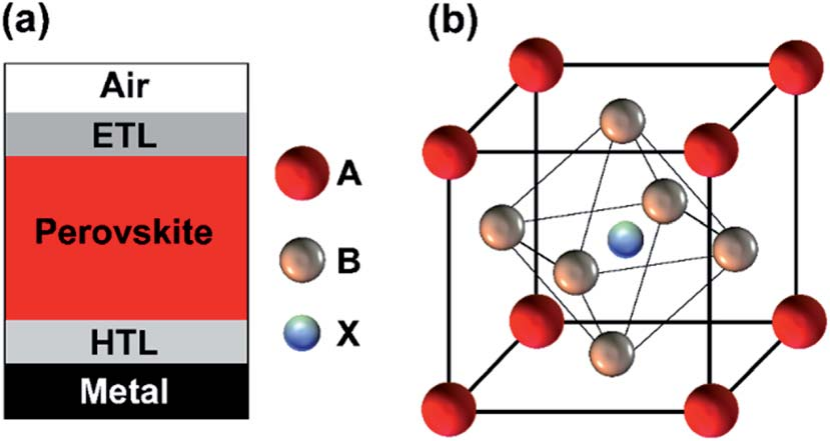
Schematic sketch of (a) a planar perovskite solar cell structure and (b) a perovskite crystal structure.

In a typical single-junction PSC, the perovskite absorber is sandwiched between an electron transport layer (ETL) and the hole transport layer (HTL) as depicted in Fig. 1(a). The selecting transport materials are vital in achieving high ECEs. However, the transport layers have to fulfill several important requirements.^14^ The charge transporting/blocking layers must exhibit a suitable work function, a high lateral conductivity, low absorption losses, and ideally support the incoupling of light in the solar cell.^14,15^ Recently, promising results have been demonstrated by using inorganic metal oxides (zinc oxide (ZnO), nickel oxide (NiO)) as charge transport layers for the implementation of PSCs.^9,16,17^ The materials fulfill several of the requirements. The metal oxides can be prepared by spin coating from nanoparticle solution, physical vapor deposition (PVD), chemical vapor deposition (CVD), and atomic layer deposition (ALD).^18,19^ Among them, ALD is a unique deposition technique for the precision-controlled deposition of high-quality metal oxide films for optoelectronic device applications.^19,20^ ALD is a specific pulse-mode CVD method which allows for self-limiting film growth. ALD films are characterized by large-area uniformity, high film conformity, sub-monolayer atomic precision thickness control, pinhole or defect-free films over large substrate areas, and most importantly low-temperature deposition. Due to the aforementioned advantages, ALD has gained considerable interest in the solar cell community.^19–22^ Recent studies reveal that the long-term stability of solar cells has been notably enhanced by encapsulating them with an ALD-grown Al_2_O_3_ protective layers.^23^

The manuscript is divided into two major parts. In the first part, aluminum-doped zinc oxide (AZO) layers were prepared by ALD as potential charge transporting/blocking layers. In the following, we refer to ZnO and AZO films prepared by ALD. ZnO refers to ALD films without nominal aluminum doping, while AZO refers to intentionally aluminum-doped ZnO films. The contact layers will be used as charge transport/blocking layers of PSCs. Herein, we focus on a planar PSC in substrate configuration. ALD-grown AZO and ZnO will be used as a potential front transparent contact and part of the rear contact, respectively. The front contact is imperative for the implementation of efficient PSC. In the second part of the manuscript, the optics of single-junction PSCs and perovskite/silicon TSCs was studied. The optics was investigated with the aid of finite-difference time-domain (FDTD) optical simulations, where experimentally realized material properties of metal-oxides were used as input parameters. Based on the electromagnetic field distribution and power density distribution within the solar cell, the quantum efficiency (QE), and short-circuit current density (*J*_SC_) was calculated. The calculated short circuit currents are compared to upper theoretical limits for the *J*_SC_. In the case of the perovskite/silicon TSC a potential upper ECE is estimated.

The design of PSC and the used optical materials are described in Section 2. The experimental details on how to prepare metal oxide contacts are presented in Section 3. Material characterization results are illustrated and explained in Section 4. The optics in PSCs is discussed and results are presented in Section 5 and guidelines are provided to maximize the *J*_SC_ and the ECE. Finally, the results are summarized in Section 6.

## 2. Material properties and device geometry

The crystal structure of perovskites is depicted in Fig. 1(b). The hybrid organic–inorganic metal halide-based materials can be defined as described by the chemical formula ABX_3_ where A and B are being cations, and X is an anion.^24^ In this study, methylammonium lead-iodide (MAPbI_3_) perovskite material (bandgap of ∼1.6 eV). In a typical PSC structure, the perovskite absorber is sandwiched between an electron transport layer (ETL) and the hole transport layer (HTL) as depicted in Fig. 1(a). Herein, metal oxides are used as potential HTL and ETL. In the current study, we used high-quality AZO and ZnO as potential front and rear contacts, which were prepared by ALD at low growth temperatures of less than 200 °C.

Perovskite and metal oxides have comparable refractive indices ranging from 2.1–2.5, which helps to minimize the reflection losses. The optical constants used for the simulations are provided in the ESI (Fig. S1†), where the complex refractive index of MAPbI_3_ was adapted from the literature.^25^ A planar PSC structure is investigated in the current study as depicted in Fig. 7(a), where a 50 nm thick ALD-grown AZO is used as an electron transport/hole blocking layer (ETL). A MAPbI_3_ perovskite absorber is sandwiched between the front and back contact, where the thickness of the perovskite is varied from 100 nm to 400 nm. A double NiO/ZnO layer is used as a hole transport or an electron blocking layer (HTL), where a 50 nm thick ALD-grown ZnO is combined with a very thin NiO. The NiO/ZnO double layer acts as a tunneling junction.^26^ The double NiO/ZnO layer exhibits a suitable work function, high lateral conductivity, and low absorption losses.^9,27^ Similar combinations of metal oxides have been used to realize contacts of highly efficient PSCs.^26,28^ A thick aluminum (Al) is utilized as a back reflector. For the case of perovskite/silicon tandem devices, the double layer of NiO/ZnO acts as an interconnector and tunnel junction.^16,29–31^ The fabrication of the perovskite solar cell (PSC) on top of the heterojunction silicon solar cell is complex, which requires a detailed understanding of optics and interface engineering. The perovskite material system and silicon exhibit different refractive indices (*n*_perovskite_ ≈ 2, *n*_perovskite_ ≈ 4), so that an optically matched layer must be introduced between the top and bottom cells to achieve efficient light incoupling. In our previous study, we have shown that the efficient coupling of light in the silicon bottom solar cell can be achieved by an optical matching layer.^9^ The optics of such a layer can be described by effective media theory. Further details are provided in ref. 9.

In the top PSC, NiO was used as a potential hole transporting/electron blocking layer (HTL) with a high work function.^16,29^ Nevertheless, NiO exhibits low conductivity and low charge carrier mobility (<1 cm^2^ V^−1^ s^−1^).^16,29,30^ The conductivity of a NiO film can be increased by doping, however, it decreases the optical transmission distinctly, leading to absorption losses. Consequently, a drop in QE and *J*_SC_ of the PSC is observed. Henceforth, we replaced the NiO layer by a double NiO/ZnO layer. A very thin NiO layer provides efficient hole injection and electron blocking, while the distinctly thicker ZnO exhibits high conductivity and high optical transparency.^28,31,32^ The NiO/ZnO double-layer acts as a tunneling junction between the top and bottom solar cell. Experimental results of TSCs with high ECE using NiO/ZnO double are provided in the literature.^10,33^ All investigated PSC structures are assumed to be realized in substrate configuration, which allows for integrating the solar cell on top of a c-Si bottom solar cell to form a perovskite/silicon TSC.

## 3. Experimental details

**Fig. 2:**
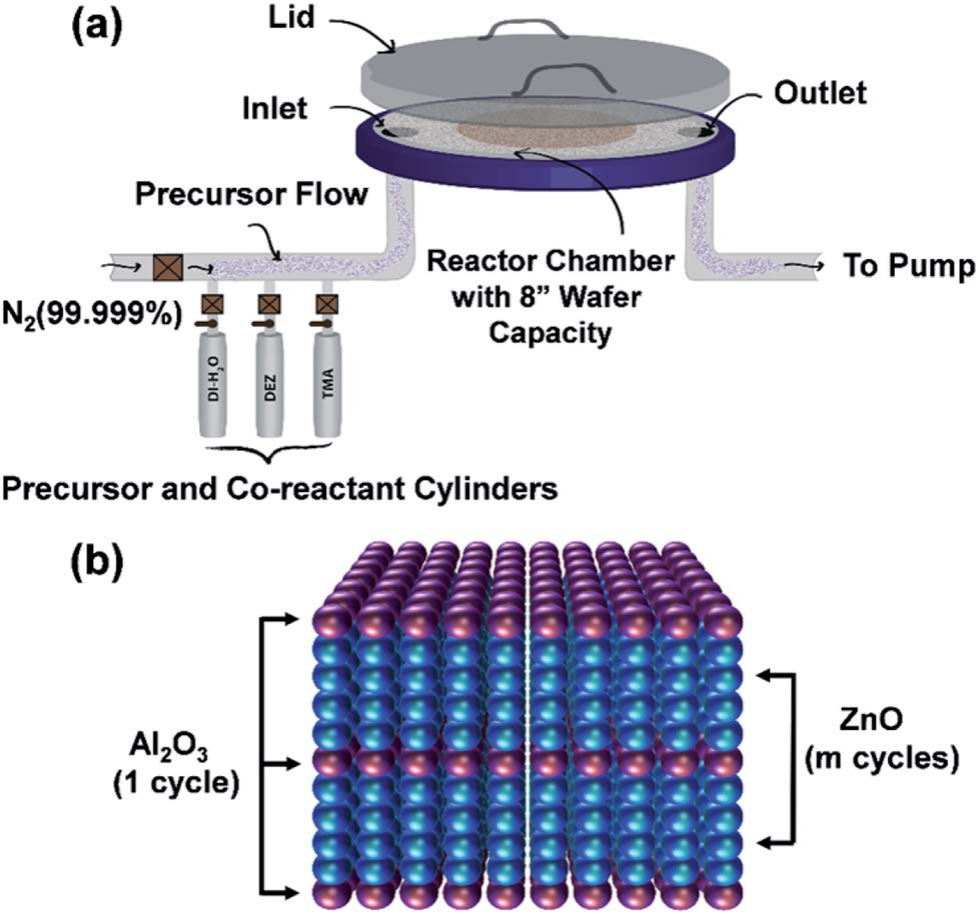
(a) ALD thermal system description and substrate arrangement (b) schematic of the deposition process for the growth of AZO.

ALD technique allows depositing a wide range of metal oxides, such as TiO_2_, ZnO, SnO_2_, NiO, *etc.* for optoelectronic applications.^19^ For the ALD process of a metal oxide compound, the gaseous vapor from a chemical precursor usually in liquid or solid form is used in conjunction with an oxidizing co-reactant such as H_2_O, O_2_, or O_3_, for the periodic surface reactions separated by purging cycles so that film growth reactions occur only on the film surface in a self-limiting manner.^20,34,35,45^ A repetitive supply of precursors leads to the deposition of the desired film at a growth rate less than a single monolayer, which ensures the formation of a pinhole defect-free film on the substrate surface. The optoelectronic properties of deposited films mainly depend on the deposition temperature, pressure, choice of precursors, and the total number of utilized ALD cycles.^19,21,34^ In this study, a thermal ALD system (Okyay Technologies, Inc.) is utilized to prepare ZnO and AZO films on silicon and glass substrates. Description of the used ALD system and substrate loading is illustrated in Fig. 2(a). Detailed explanations of the deposition process and film characterizations will be discussed in the following subsections.

### 3.1 Deposition of metal-oxides

In this study, ZnO and AZO films were deposited thermally by ALD, where silicon (100) and glass (Corning 7059) were used as substrates. Throughout the experiments, diethylzinc ((Zn(C_2_H_5_)_2_), DEZ, Sigma-Aldrich) and trimethylaluminum (TMA, 99.999%, Strem Chemicals, Inc.), and deionized (DI) water (H_2_O) were used as precursors and oxidant. Nitrogen (N_2_, 99.999% purity, Airgas) was used as both carrier and purging gas. In all experiments, precursors and water oxidants were kept at room temperature (22–24 °C) in the lab. The substrate temperature was varied from 100 °C to 200 °C with an interval of 50 °C. The base pressure of the chamber was kept below ∼100 mTorr throughout the experiments. Up to 500 ALD cycles were performed to investigate the layer by layer film growth in the self-limiting growth regime. Before loading the substrates into the ALD chamber, silicon and glass substrates were ultrasonically cleaned with solvents (acetone and isopropanol) and deionized water in a sequence for 2 minutes, next dried with nitrogen flow. Prior to start, the deposition of zinc-oxide films, standard alumina (Al_2_O_3_) film was grown as a reference sample, where TMA and water were used as precursors and co-reactant. The deposited alumina films exhibited excellent uniformity and high quality in terms of their optical properties (refractive index) and surface morphology.

#### 3.1.1 Deposition of zinc-oxide (ZnO) film

DEZ and DI water were selected as precursor and oxidant for the deposition of ZnO films on silicon and glass substrates. The precursor and oxidant were alternately supplied into the ALD reactor by using external intrinsic vapor pressures. The ALD reaction mechanism for the ZnO film growth can be defined by the following equation:

1 Zn(CH_2_CH_2_)_2_ + H_2_O → ZnO + 2C_2_H_6_

The deposition of ZnO film is a quite straight forward process, where a single ALD reaction cycle consists of a 15 ms exposure to DEZ, followed by a 10 s for N_2_ purge, a 15 ms exposure to H_2_O, and then another 10 s N_2_ purge to remove reaction by-products and an excess amount of precursor vapor entirely. The total number of ALD reaction cycles ranged from 50 to 500 allowing to choose the film thickness within ∼9 to 90 nm.

#### 3.1.2 Deposition of Al-doped zinc oxide (AZO) film

Similar substrates (silicon and glass) were used to deposit AZO films, where the substrate temperature was kept constant to 150 °C. DEZ and DI water vapor served as zinc and oxygen precursors, where they alternatively introduced in the ALD reactor through the chamber inlet using N_2_ carrier gas at a flow rate of 20 sccm. TMA and DI water were selected as aluminum and oxygen precursors for the Al_2_O_3_ growth, which is used to dope the ZnO film. In this experiment, the pulsing time of precursors (DEZ and TMA) and oxidant (H_2_O) was kept constant at 15 ms, where purging flow time was 10 s for all cases. The ALD growth of AZO can be described by the following expression (eqn (2)).

2 [A × (DEZ + H_2_O) + B × (TMA + H_2_O)] × C

where [A × (DEZ + H_2_O) + B × (TMA + H_2_O)] represents one supercycle and C is the number of supercycles for the AZO film deposition. The supercycle was repetitively performed until obtaining the expected film thickness, where multiple (m) ZnO cycles repeated for one (1) Al_2_O_3_ cycle as illustrated schematically in Fig. 2(b). Henceforth, the number of DEZ/H_2_O cycles carried out for the AZO film deposition depends on the deposition cycle ratio ZnO : Al_2_O_3_. In this experiment, the deposition cycle ratio varied from 15 : 1 to 50 : 1 to realize the better AZO film having low resistivity (high conductivity) and high transparency. A similar number of ALD cycles (up to 500) was performed for the AZO deposition, where the total number of ALD cycles was determined by adding individual ZnO and Al_2_O_3_ cycles. It is noted that the pulse time of individual precursor flow has been optimized to the minimum value (15 ms) which is notably lower than the reported value in the literature.^17,20,34^ Hence, the proposed deposition process might result in high quality and at the same time low-cost device layers.

## 4. Materials characterization

**Fig. 3:**
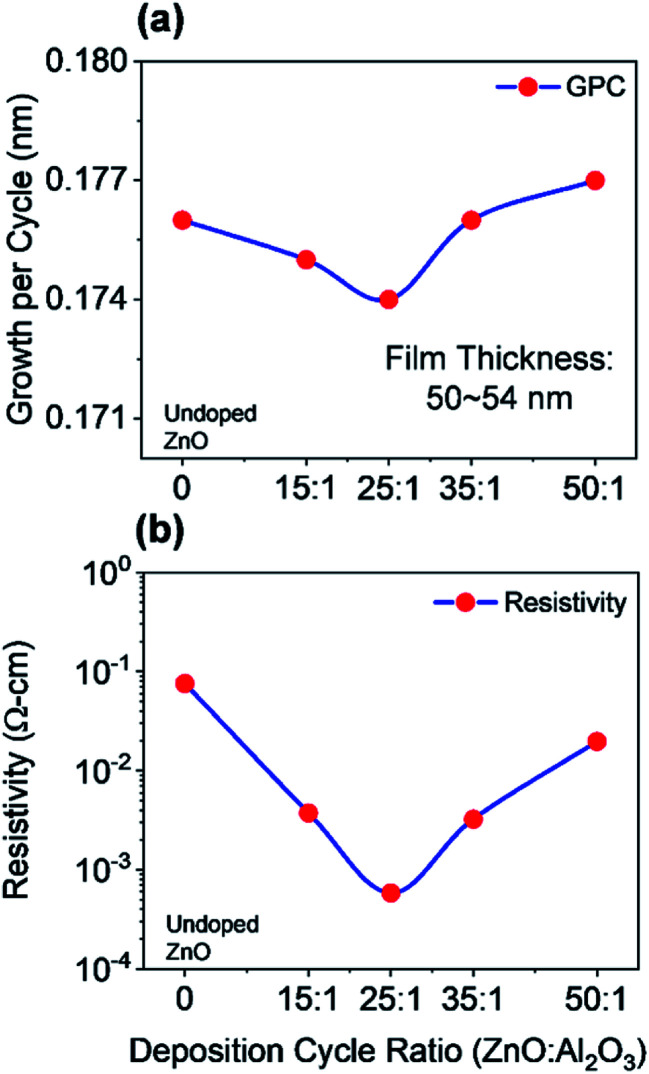
(a) Growth per ALD cycle and (b) measured resistivity as a function of the deposition cycle. ZnO and AZO films on silicon substrates were used to determine the GPC, whereas films on glass substrates used for the determination of resistivity.

As a part of this study, different physical properties of the deposited films have been characterized by using several techniques, which lead to realizing the better contact layers for PSCs. The electrical and optical characterizations were performed on the films deposited on silicon substrates, where films deposited on glass substrates were used for the morphological and structural analyses. All measurements were performed at room temperature (22–24 °C). To begin the characterization process, we started to study the growth mechanism of undoped ZnO films for multiple ALD reaction cycles which were deposited at 150 °C. The growth property of deposited films was determined by the *ex situ* multi-wavelength ellipsometry measurement (FS-1 Multi-wavelength Ellipsometer, Film Sense, LLC). This ellipsometry allows determining the wavelength-independent refractive index, where we have obtained the expected refractive index (∼2) for deposited ZnO films. The film thickness varies with changing the number of ALD cycles, where ZnO film thickness ranged from ∼9 nm to ∼88 nm for 50 to 500 ALD cycles. The growth per cycle (GPC) for individual ZnO films varies from ∼0.177 nm to ∼0.175 nm. The thickness of the deposited film is increased linearly due to an almost constant GPC, which demonstrates the self-limiting behavior of the ALD growth process. Additionally, we also measured the growth rate (∼0.1 nm per cycle) and refractive index (∼1.6) of previously deposited Al_2_O_3_ film which will be used as a reference to determine the doping percentage. Next, we studied the growth of AZO film for different deposition cycles. In general, the growth rate of AZO films is relatively lower than the growth rate of ZnO which might occur due to the insufficient energy of chemical reactions. The growth rate of deposited AZO films decreases with the increase of the Al percentage up to 25 : 1 deposition cycle and then starts to increase with a further increase of dopant. Typically, the minimum growth rate is useful to achieve a uniform and good quality film. Fig. 3(a) shows the growth per cycle of AZO for different deposition cycles along with undoped ZnO, where the total number of ALD cycles was 300 considered. The corresponding film thickness is varied from ∼50 nm to ∼54 nm with an average growth rate of ∼0.18 nm per cycle. Our calculated GPC has a comparable value which was determined by other research groups.^32,34,36^ The nominal Al percentage in the AZO film can be determined by the following formula (eqn (3)).^37^3
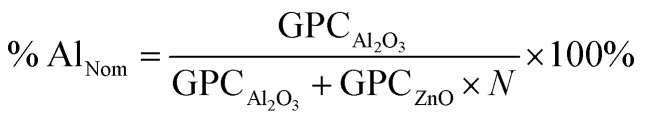
where GPC_Al_2_O_3__ and GPC_ZnO_ represents the growth rates of Al_2_O_3_ and ZnO, respectively, where *N* indicates the number of supercycles.

According to the eqn (3), the nominal Al percentage of AZO film, which has a comparatively low GPC (25 : 1 deposition cycle), was calculated to 2.14%. One of the major optoelectronic applications of ZnO films is to use as a transparent contact, where both high conductivity (low resistivity) and high transparency are required. In PSC, the front contact should be thin enough to minimize series resistance so that optical losses are minimized. The thinner front contact should also provide an efficient charge injection. Since deposited AZO and ZnO films will be used as potential front and back contacts in this study for the implementation of PSCs, it is necessary to evaluate the performance as a contact layer by understanding the electrical properties of deposited films. The electrical characterization of deposited AZO and ZnO films was carried employing four-point probe measurements by using the Alessi CPS system at room temperature. The system allows determining the sheet resistance of the film, which can be further multiplied with film thickness for the realization of a resistivity. To avoid spatial resistivity distribution, films were deposited on glass substrates which patterned in squared-shaped (1 cm × 1 cm) following the van der Pauw geometry. Fig. 3(b) shows the measured resistivity of AZO and ZnO films as a function of the deposition cycle ratio. In the case of undoped ZnO, the resistivity is 7.56 × 10^−2^ Ω cm.

As aluminum incorporated into ZnO films, the resistivity of AZO decreases and reaches the lowest value of 5.84 × 10^−4^ Ω cm for a 25 : 1 deposition cycle ratio. Further doping of Al leads to a slight increase in the resistivity to 3.74 × 10^−3^ Ω cm for the 15 : 1 deposition cycle ratio. The lowest resistivity was achieved for 25 : 1 AZO which is lower than the results reported in the literature.^21,34,37^ The deposition was carried by controlling the number of supercycles consisted of DEZ/H_2_O : TMA/H_2_O cycle ratio 25 : 1, as explained in the deposition process.

**Fig. 4:**
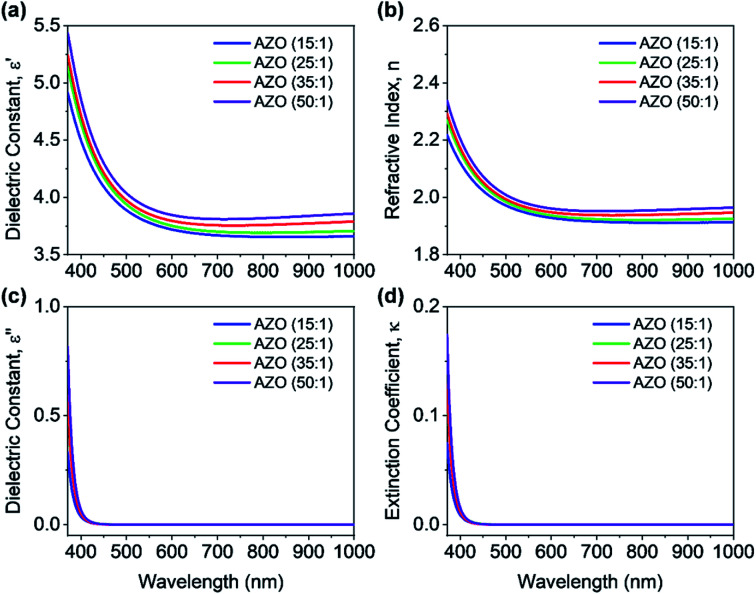
(a) Real and (b) imaginary part of the complex dielectric function of AZO films as a function of the incident wavelength. (c) Real and (d) imaginary part of the complex refractive index of AZO films as a function of the incident wavelength. Films were grown on silicon substrates for the optical measurement.

To start with the optical characterization, the spectroscopic ellipsometry (J.A. Woollam Co. Inc. Spectroscopic Ellipsometer) measurements were performed on AZO and ZnO films deposited on silicon substrates. The wavelength ranged from 371 nm to 1000 nm at three incident angles of 60°, 65°, and 70° for the measurements. The ellipsometry techniques allow determining complex dielectric constants and refractive index of deposited films. Fig. 4 shows the measured optical constants of AZO films, where it is found that complex dielectric constant and refractive index of 50 : 1 AZO is almost like undoped ZnO. Hence, only the optical constants of AZO films are shown in Fig. 4, where the deposition cycle ratio is varied from 15 : 1 to 50 : 1. A blue or UV shift is observed in the optical constants by increasing the Al doping percentage since Al impurity can act as effective n-type donors for generating free carriers. The doping concentration of DEZ/H_2_O can increase the free carrier concentration that reduces the refractive index of AZO film. Fig. 4(d) shows the extinction coefficient of AZO films for various deposition cycle ratios. The extinction coefficient is close to zero for the entire wavelength range 400–1000 nm, which indicates the transparency within this wavelength range. The bandgap of the deposited AZO films is approx. 3.1 eV.

**Fig. 5:**
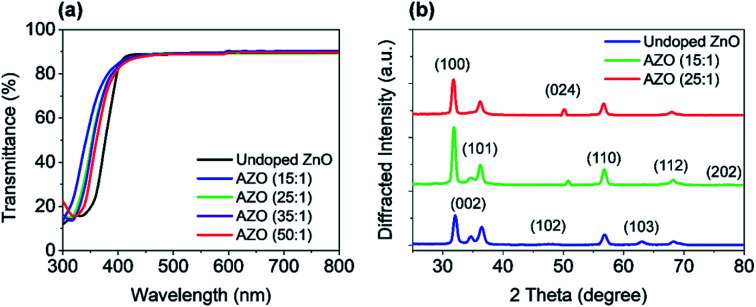
(a) Optical transmittance spectrum of ZnO and AZO films with various deposition cycles, grown on glass substrates. (b) X-ray diffraction patterns of ZnO and AZO films with various deposition cycles, grown on Si substrates.

The optical transmission of the AZO and ZnO films on a glass substrate is shown in Fig. 5(a) for the wavelength range 300 nm to 800 nm. The deposition cycle ratio of AZO is varied from 15 : 1 to 50 : 1. The average transmission of the deposited samples is approx. 90% in the visible wavelength region (400–700 nm). The UV shift of absorption edge (300–400 nm) has been found with increasing Al doping level, which is very consistent with the results measured by the spectroscopy ellipsometry. Such ALD growth films exhibit great potential as a transparent conductor for PSC applications, which can be further applied to the implementation of transparent solar cells.

For the structural analysis, X-ray diffraction (XRD) measurements were done on deposited ZnO and AZO films. The crystalline structures were performed for the deposited films by using Rigaku SmartLab automated multi-purpose X-ray Diffractometer with CuK_α_ radiation (*λ* = 0.154 nm) at room temperature. The XRD spectra were measured on all samples in the two-theta (2*θ*) angular region between 25° to 80°. The XRD patterns of ZnO and AZO with the deposition cycles of 15 : 1 and 25 : 1 is shown in Fig. 5(b), where comparison of XRD pattern of only AZO films for various deposition cycle ratios are presented in the ESI (Fig. S2†).

Besides, temperature-dependent XRD patterns for the 25 : 1 (optimal cycle ratio) AZO film is shown in the ESI (Fig. S3†). All XRD patterns exhibit the polycrystalline structure with orientation along different planes. These planes are (100), (002), (101), (102), (110), (103), and (112) of undoped ZnO, which confirm that films are polycrystalline wurtzite structure. Major diffraction peaks ((100), (002), (101), and (110)) are observed at 31.8°, 34.5°, 36°, and 56.5° in the undoped ZnO film. Nevertheless, at 34.5° diffraction peak (002) has a less intensity which disappears by Al doping, where (002) is completely missing in 15 : 1 AZO case. On the other hand, peak (100) at 31.8° becomes more dominant after increasing the Al doping level, which is similar to the results reported in the literature. Moreover, increased doping causes to introduce new peaks which are (024) and (202).

**Fig. 6:**
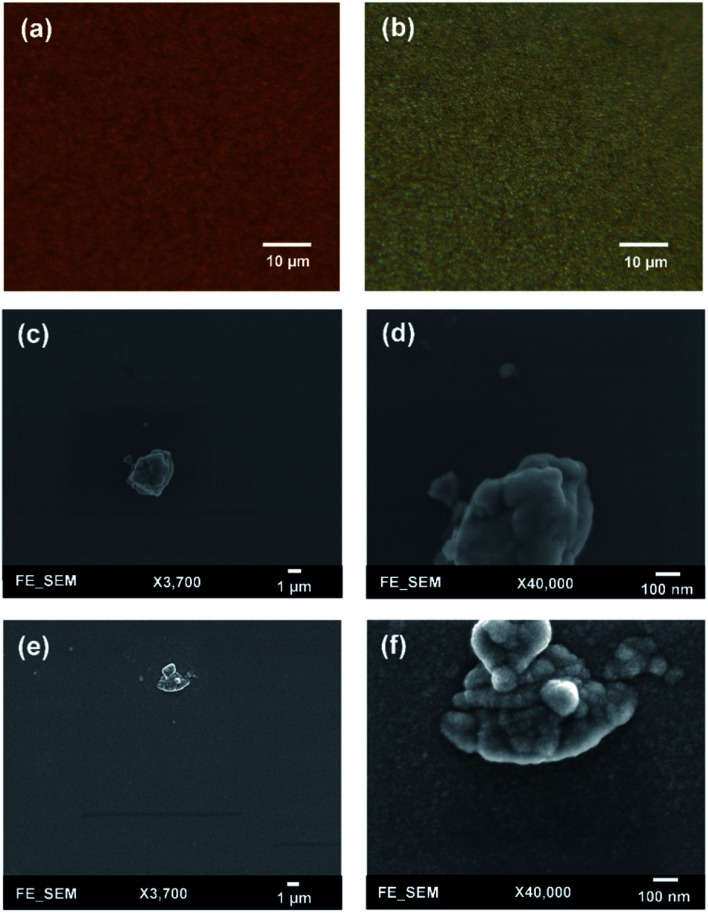
Optical microscopic images of (a) 25 : 1 AZO and (b) undoped ZnO. Top view of SEM micrographs of (c and d) 25 : 1 and (e and f) undoped ZnO. All films were grown on silicon substrates at 150 °C deposition temperature.

To check the uniformity of deposited films, surface morphologies of AZO and ZnO films grown on a silicon substrate were studied by using optical microscopy (Leica DM 2700M) and field emission scanning electron microscopy (FESEM JEOL JSM-6335F). Surface morphologies were first investigated with optical microscopy where all films exhibit a good uniformity and no significant difference is observed between AZO and ZnO films. Fig. 6(a and b) shows optical microscopic images of 25 : 1 AZO and ZnO films for a large area. Next, surface morphologies were investigated by using FESEM, where typically all films exhibit good uniformity over the scanning area. High-resolution SEM images of ZnO and 25 : 1 AZO are depicted in Fig. 6(c and d), where film thickness varies between 50–54 nm. In low magnification images, it has been seen that all films show a great uniformity which is highly dense. For higher magnification, AZO film demonstrates a relatively smoother than undoped ZnO film. The influence of Al doping makes the ZnO film more uniform which leads to prepare a high-quality film. Generally, all samples show a smooth surface with low surface roughness which will help to improve the stability and performance of PSCs.

## 5. Optics in perovskite solar cells

**Fig. 7:**
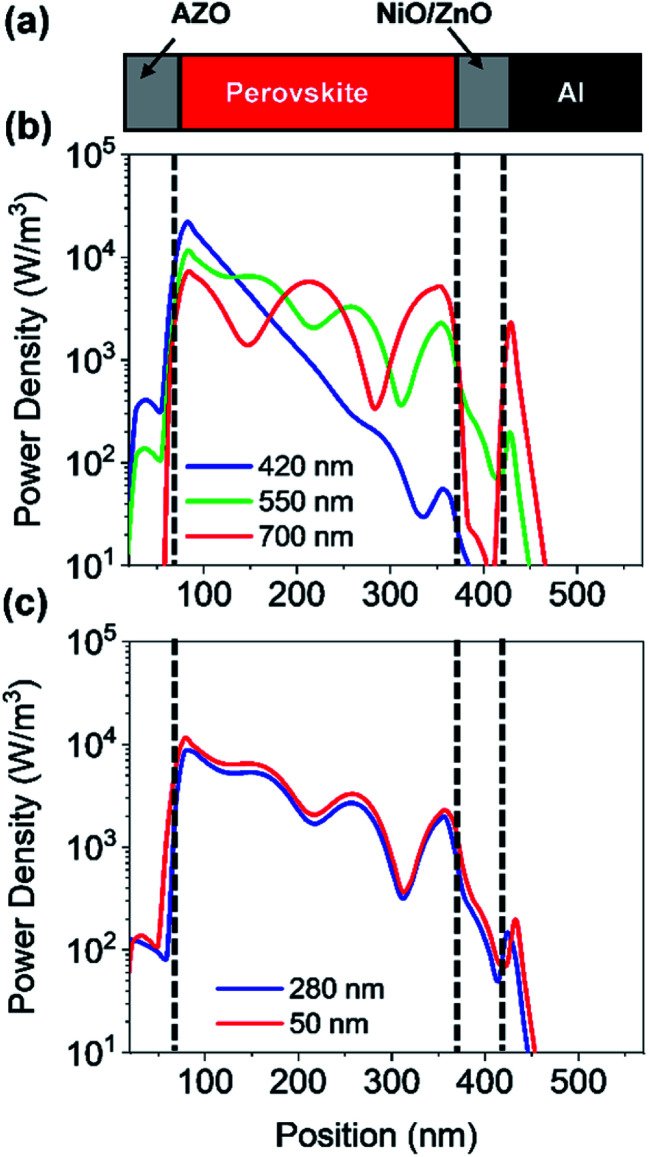
(a) Schematic cross-section of a perovskite planar solar cell with AZO front contact. (b) Power density profile of perovskite solar cell with an AZO front contact (deposition cycle ratio 25 : 1) for different incident wavelengths. (c) Power density profile of perovskite solar cell with an AZO front contact for different front contact thicknesses at an incident wavelength of 550 nm.

Three-dimensional FDTD optical simulations were performed to study the influence of the device design on the *J*_SC_ and derive an optimal device design. The complex refractive index of the materials and the geometry of the devices were used as input parameters. The optical constant of materials used for the simulations are provided in the ESI (Fig. S1†). The optical constants of the AZO films were taken from spectroscopic ellipsometry measurements as described in Section 4. Simulations are carried out for the wavelengths range from 300 nm to 800 nm, where the circularly polarized incident wave has an amplitude of 1 V m^−1^. Simulations of the tandem solar cell were carried out for wavelengths ranging from 300 nm to 1200 nm. The collection efficiency of solar cell absorbers is assumed to be 100%. This assumption is valid if the charge carrier diffusion length is larger than the thickness of the absorber. The measured diffusion length is large enough for MAPbI_3_ (1.7 μm for electrons and 6.3 μm for holes), while the thickness of our perovskite solar cells is smaller than 400 nm.^38^ The calculated QE of the solar cell represents an upper limit of the QE. The optical simulation provides the electric field distribution within the solar cell structure. The time-averaged power density within the solar cell is given by4
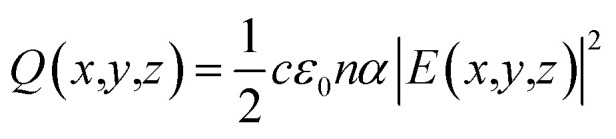
where *c* and *ε*_0_ are the speed of light in free space and permittivity of free space. *n* and *E* are the refractive index of the material and electric field distribution. *α* is the absorption coefficient. Based on the power density, the quantum efficiency of PSC is calculated by5
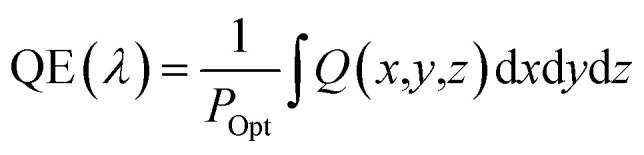
where *P*_Opt_ is the optical input power of the sun. The QE is defined as the ratio of photons absorbed by the perovskite layer divided by the total photons incident to the solar cell. The short-circuit current density can be calculated by6
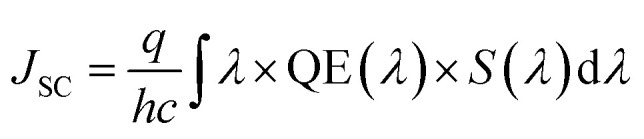
where *h* is the Planck's constant and *S*(*λ*) is the solar spectral irradiance (AM1.5 G). Photons absorbed by the perovskite layer contribute to the *J*_SC_, and photons absorbed by all other layers do not contribute to the *J*_SC_. The schematic cross-section of a PSC on a smooth substrate is shown in Fig. 7(a). The solar cell consists of an Al back reflector and a NiO/ZnO hole transporting and electron blocking layer. The back contact is followed by a perovskite layer and an AZO front contact. The optical constants of the ALD films were used for the investigation.

### 5.1 Perovskite single junction solar cells

In a first step, we have investigated the influence of the front contact on the QE and *J*_SC_ of perovskite single junction PSCs. The sheet resistance of the front contact was kept constant to 80 ohms, while the doping concentration was varied.^39^ Hence, the AZO film thickness must be adjusted to keep the sheet resistance of the front contact constant, which is given by *R*_S_ ≅ *d*/(*q* × *N*_D_ × *μ*_e_), where *d* is the film thickness, *q* the elementary charge, *N*_D_ the donor doping concentration and *μ*_e_ the electron charge carrier mobility. The charges are collected by a metal front contact grid printed on the solar cell. For the given sheet resistance, the thickness of the AZO film was varied from 50 nm to 1690 nm. Fig. 7(b) exhibits the power density profile of a solar cell with an AZO thickness of 50 nm under monochromatic illumination of 420 nm, 550 nm, and 700 nm. At 420 nm wavelength, most of the incident photons are absorbed within the first 150 nm of the perovskite film.

**Fig. 8:**
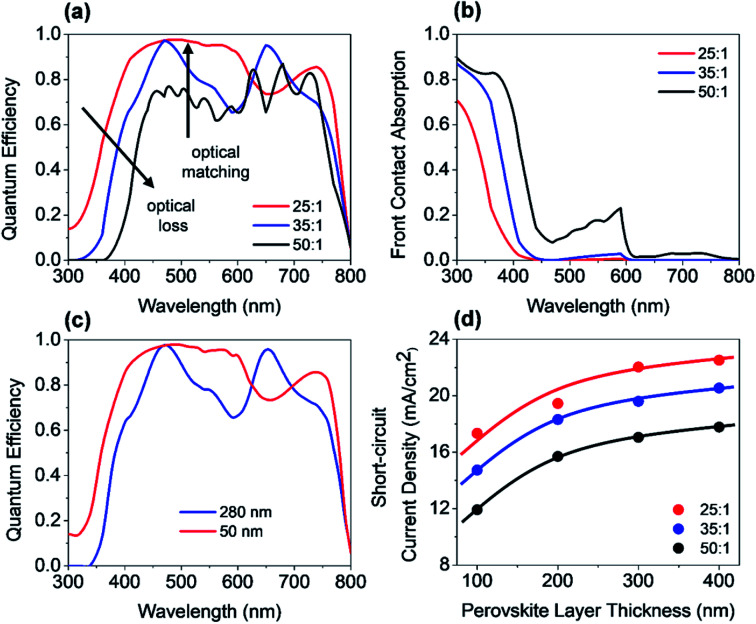
(a) Quantum efficiency and (b) front contact absorption of PSC with equal front contact sheet resistance but the different thickness and doping concentration. (c) Quantum efficiency of PSC with equal doping concentration but different thicknesses. (d) Short-circuit current density of PSC with equal front contact sheet resistance but the different thickness and doping concentration as a function of perovskite absorber thickness.

At 700 nm, a large fraction of the incident light reaches the back contact, where it is reflected, and a standing wave is formed inside the solar cell due to constructive and destructive interference as shown in Fig. 7(b). The QE of solar cells with equal sheet resistance but different doping concentration and thickness is shown in Fig. 8(a). The solar cell with a front contact thickness of 50 nm exhibits the highest QE. An increased QE is observed for short-wavelength due to low absorption of the thin AZO layer.

Furthermore, an increased QE is observed for the spectral range from 400 nm to almost 600 nm. The front contact acts as a quarter wavelength thick anti-reflection coating for an incident wavelength of 500 nm. The thickness of an anti-reflection coating is commonly given by *λ*/(4 × *n*_ARC_), where *λ* is the wavelength at which the reflection is minimized, and *n*_ARC_ is the refractive index of the anti-reflection coating. In our case, the thickness of the anti-reflection layer is given by7
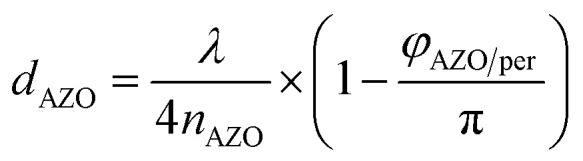
where *n*_AZO_ is the refractive index of the front contact and *φ*_AZO/per_ is a phase shift introduced by the AZO/perovskite interface.

The phase shift is caused by the large extinction coefficient of the perovskite layer. The phase shift is calculated to be approx. π/4. Without the phase shift, the thickness of the front contact would have to be 65 nm to achieve anti-reflection properties at 500 nm. The phase shift at the AZO/perovskite interface leads to a reduction of the front contact thickness down to 50 nm. Decreasing the doping concentration of the front contact leads to an increased thickness, which causes an increased absorption loss of the front contact. Furthermore, the front contact does not act as an anti-reflection coating anymore. Multiple interferences in the front contact are visible in the QE for the front contact doping concentration of 50 : 1 and a thickness of 1690 nm. A comparison of a solar cell with thin and thick front contact is provided in Fig. 7(c). The plot shows the power density profile for an incident wavelength of 550 nm and a front contact thickness of 50 nm and 280 nm. The shape of the power density profile is not affected by the different front contact. Only the incoupling of light by the front contact is affected. Moreover, the influence of different front contact thicknesses on the *J*_SC_ was investigated, which is shown in Fig. 8(d). The *J*_SC_ is varied from 17.3 mA cm^−2^ to 22.5 mA cm^−2^ for perovskite thicknesses from 100 nm to 400 nm, respectively.

The *J*_SC_ is enhanced by 45% for 100 nm thick solar cells and 26% for a solar cell with a thickness of 400 nm. The *J*_SC_ of the PSC with an anti-reflection coating is comparable to the PSC with a textured front contact.^15^

### 5.2 Perovskite/silicon tandem solar cells

**Fig. 9:**
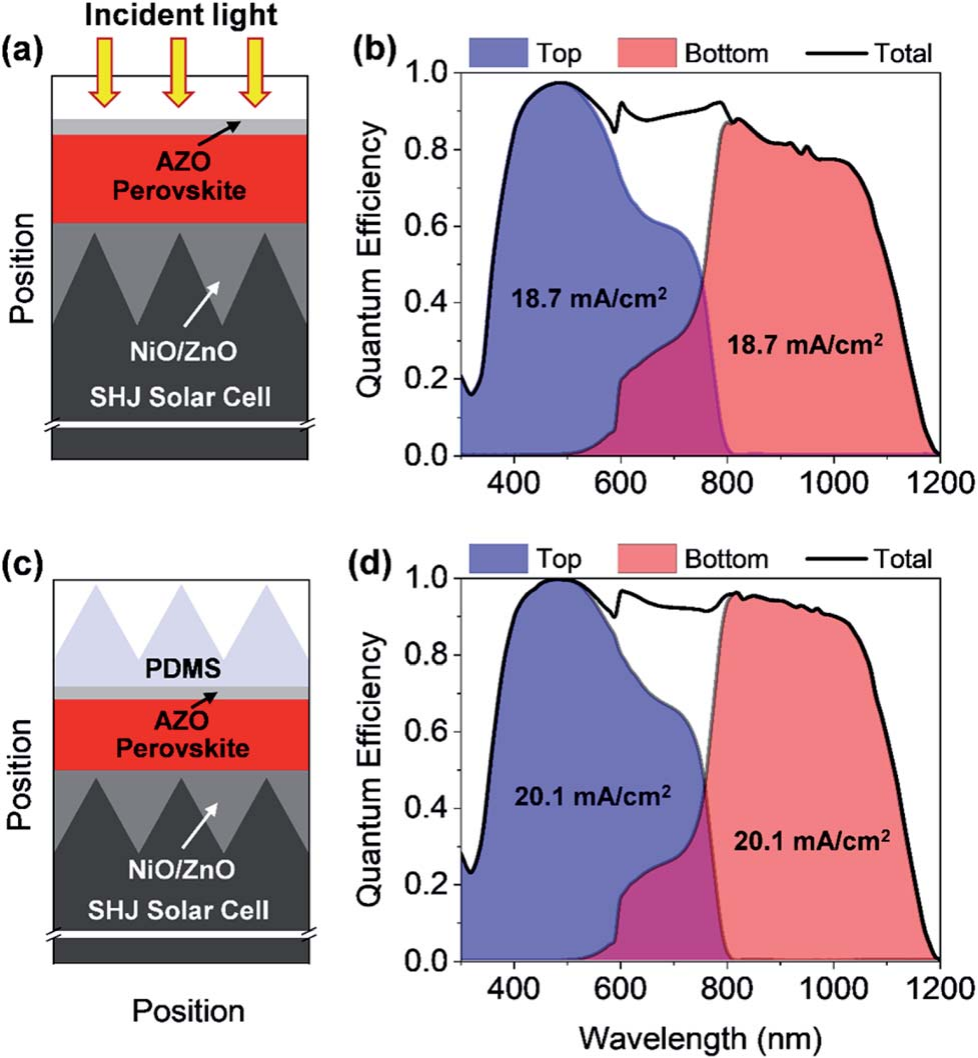
Schematic diagram of the perovskite/silicon tandem solar cells with (a) flat AZO front contact (c) flat AZO front contact and pyramidal textured PDMS coupler. (b and d) The corresponding quantum efficiency for the top and bottom cells, and the total quantum efficiency are shown.

In this section, the optics of a perovskite/silicon TSC is discussed. The optics of a perovskite thin-film solar cell are very different from the optics of a silicon solar cell and the integration of a PSC and a c-Si solar cell is complex. The optical properties of silicon and perovskites are very different. Silicon is an indirect bandgap material with a rather low absorption coefficient close to the band edge, while the refractive index of silicon is high. Efficient light management is essential for achieving high *J*_SC_s and ECEs. The light must be efficiently coupled in the solar cell and the light must be diffracted and refracted to enhance the optical path length in the solar cell. The perovskites material system exhibits a penetration depth, which is commonly smaller than the thickness of the solar cell. Hence, only the coupling of light in the solar cell is of major importance. The PSC is formed on a planarized silicon heterojunction (SHJ) solar. The silicon solar cell is planarized to allow for the realization of the planar top solar cell. The bottom solar cell must be textured to allow for efficient coupling of the light in the bottom solar cell. The planarization is achieved by filling the valley of the surface texture by zinc oxide. The zinc oxide/silicon texture acts as an optical matching layer. The PSC is formed on the optical matching layer. First, we have investigated a TSC with a thin planar AZO layer as a front contact as shown in Fig. 9(a). The thickness of the thin front ZnO was set to 50 nm.

To determine the QE and *J*_SC_ of the solar cells, the optical wave propagation must be rigorously simulated. However, the thickness of the TSC is distinctly larger than the wavelength of the incident light, so that a rigorous simulation is complex and computationally time-consuming. Hence, a hybrid approach has been adapted to study the perovskite/silicon TSC, where a record SHJ solar cell is considered as a bottom cell.^40^ A detailed discussion about the hybrid method for the implementation of perovskite/silicon TSC is provided in the previous study.^9^ To determine the QE of the bottom solar cell, the light transmitted by the PSC is calculated. The calculated QE of the perovskite/silicon TSC is shown in Fig. 9(b). Next, the *J*_SC_ of both cells is calculated. The calculated *J*_SC_ of the individual cells must be matched for realizing a high-efficiency perovskite/silicon TSC. In the current study, the perovskite absorber thickness was adjusted until a matched *J*_SC_ is achieved. Matching of the *J*_SC_s is achieved for a perovskite layer thickness of 280 nm. Both cells provide a *J*_SC_ of ∼18.7 mA cm^−2^. This corresponds to 81% of the upper theoretical *J*_SC_ for a TSC with a silicon bottom absorber, which is calculated to be 23 mA cm^−2^. In this case, all photons with photon energy larger or equal to the bandgap of the absorber of the bottom diode contribute to the matched *J*_SC_.

By determining all optical losses, it is found that the QE and the *J*_SC_ can be further improved if the front reflection is minimized. The anti-reflection coating is optimized for the top but not the bottom solar cell. An improved incoupling can be achieved by placing a pyramid texture on the solar cell that allows for a broadband light incoupling.^41^ The schematic of a perovskite/silicon TSC by using a PDMS layer is shown in Fig. 9(c) and corresponding QE is illustrated in Fig. 9(d). It has been proposed by several authors to place a pyramidal textured polydimethylsiloxane (PDMS) stamp on the planar solar cell.^42–44^ The stamp can be simply fabricated by taking a replica of anisotropically etched silicon wafer.^42–44^ The period and height of the pyramid are assumed to be 450 nm and 300 nm, respectively. The detailed descriptions of how to realize a pyramidal texture PDMS is provided in the literature.^42–44^ The PDMS film has a refractive index of approx. 1.4, while the ZnO layer and the perovskite layer exhibit a refractive index ranging from 2.1 to 2.5. An improved incoupling of light is predominantly observed for long wavelengths which can be seen from the QE plot in the ESI (Fig. S4†). A further reduction of the reflection losses could be achieved by reducing the refractive index difference between the PDMS pyramid texture and the ZnO/perovskite layer. The thin ZnO layer has only a small effect on the coupling and scattering of light in the top diode. Hence the matched *J*_SC_ is increased from 18.7 mA cm^−2^ to 20.1 mA cm^−2^ for a perovskite layer thickness of 320 nm. This corresponds to 87% of the upper theoretical *J*_SC_ for a TSC with a silicon bottom absorber. Moreover, a realistic upper bound of the ECEs can be estimated for the proposed perovskite/silicon TSCs by combining optical simulation results with results from experimentally realized solar cells. The best silicon solar cell demonstrates *V*_OC_ and FF of ∼0.74 V and 84.9%, respectively,^40^ where PSC with a bandgap of ∼1.6 eV exhibits *V*_OC_ and FF of 1.182 V and 77%.^3^ Hence, ECEs of the proposed perovskite/silicon TSCs without and with PDMS can be determined to ∼29% and ∼31.5%, respectively.

## 6. Summary

Charge transport and contact layers have a significant impact on the short-circuit current density, open-circuit voltage and energy conversion efficiency of heterojunction solar cells like perovskite solar cells. In this study, AZO and ZnO films were investigated as potential contacts of perovskite single junction and perovskite/silicon tandem solar cells. AZO and ZnO films were prepared by using ALD at low deposition temperatures. The influence of various doping concentrations on the electrical, optical and structural properties was investigated by multi-wavelength ellipsometry, spectroscopic ellipsometry, sheet resistance, UV-Vis, microscopic, SEM, and XRD measurements. Films with resistivities down to 5.8 × 10^−4^ Ω cm for the thickness of ∼50 nm, where an almost 90% optical transmittance was achieved for the spectral range 400 nm to 1200 nm. In the second part of the manuscript, the metal oxide films were used as potential front contact of perovskite single and perovskite/silicon tandem solar cells. FDTD optical simulations were performed to investigate the optics of perovskite single-junction solar cells and perovskite/silicon tandem solar cells. In the case of the single-junction solar cell, it could be shown that thin and highly doped metal oxides can be used as efficient solar cell front contacts. The film acts as an anti-reflection coating, while the optical losses of the front contact are rather low, which allows for reaching short-circuit current density of up to 22.5 mA cm^−2^ using a 400 nm thick perovskite absorber. This corresponds to 84% of the upper theoretical short-circuit current density for an absorber with equal bandgap. Furthermore, the optics perovskite/silicon tandem solar cell was investigated. The optics of a perovskite/silicon tandem solar distinctly deviate from optics of perovskite single-junction solar cells. Hence the design must account for the different requirements. Matched short-circuit current densities of 20.1 mA cm^−2^ can be achieved if an efficient coupling of the light in the top and bottom solar cell is ensured. This corresponds to 87% of the upper theoretical short circuit current for a tandem solar cell with a silicon bottom absorber. Based on the calculated short-circuit current density in combination with published experimental results and analytical calculations, a potential upper energy conversion efficiency of ∼31.5% could be realized from the perovskite/silicon tandem solar cell.

## Conflicts of interest

There are no conflicts to declare.

## Supplementary Material

RA-010-D0RA00939C-s001
