# Supplementary material for: Atomic layer deposition of metal oxides for efficient perovskite single-junction and perovskite/silicon tandem solar cells
Source: RSC Adv. 2020 Apr 14;10(25):14856–66. doi: 10.1039/d0ra00939c (PMC9052116; doi:10.1039/d0ra00939c)
Supplement: RA-010-D0RA00939C-s001 [file RA-010-D0RA00939C-s001.pdf]

Electronic Supplementary Material (ESI) for RSC Advances.
This journal is © The Royal Society of Chemistry 2020

Supplementary Information

Atomic Layer Deposition of Metal Oxides for Efficient Perovskite Single-junction and Perovskite/Silicon Tandem Solar Cells

Mohammad I. Hossain[1,2,†], Adnan Mohammad[2,†], Wayesh Qarony[1], Saidjafarzoda Ilhom[2], Deepa R. Shukla[2,3], Dietmar Knipp[4,*], Necmi Biyikli[2,*], Yuen Hong Tsang[1,*]

1.) Department of Applied Physics, The Hong Kong Polytechnic University, Hung Hom, Kowloon, Hong Kong

2.) Department of Electrical and Computer Engineering, University of Connecticut, Storrs, CT 06269, USA

3.) Department of Materials Science and Engineering, University of Connecticut, Storrs, CT 06269, USA

4.) Geballe Laboratory for Advanced Materials, Department of Materials Science and Engineering, Stanford University, Stanford, CA 94305, USA

†: Authors equally contributed to this work

*: Corresponding author

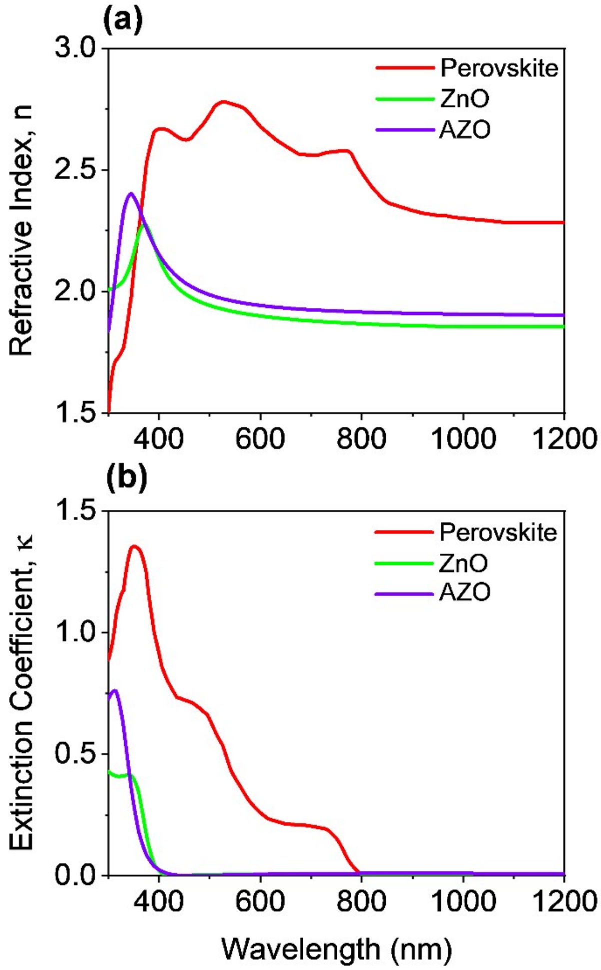

Fig. S1 (a) Refractive index and (b) extinction coefficient of perovskite ($MAPbI_3$), ZnO, and AZO for the realization of perovskite solar cells.

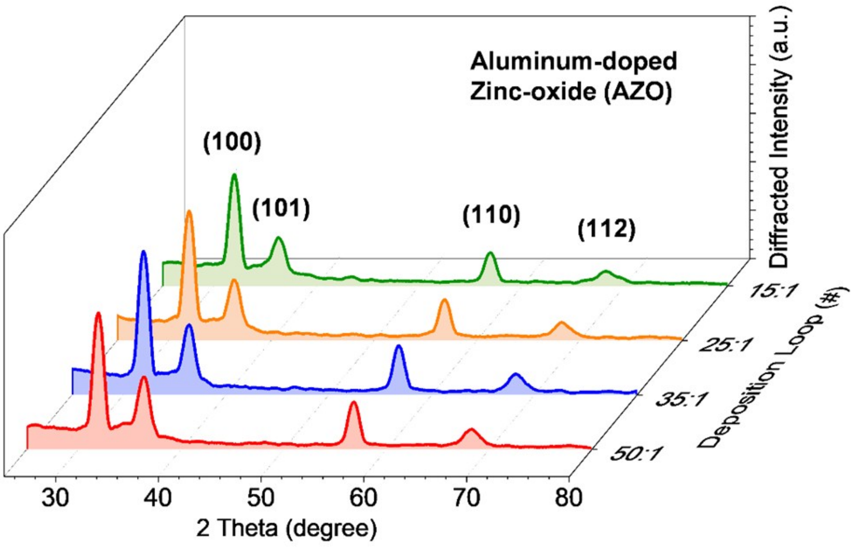

Fig. S2 X-ray diffraction patterns of AZO films with various deposition cycles, grown on Si substrates.

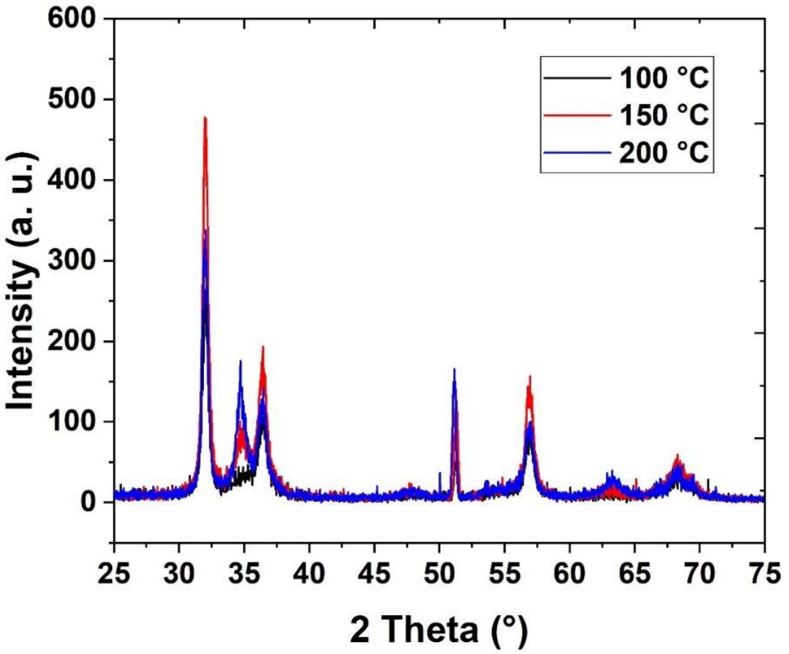

Fig. S3 X-ray diffraction patterns of AZO films with various deposition temperatures, grown on Si substrates. The deposition cycle ratio is 25:1.

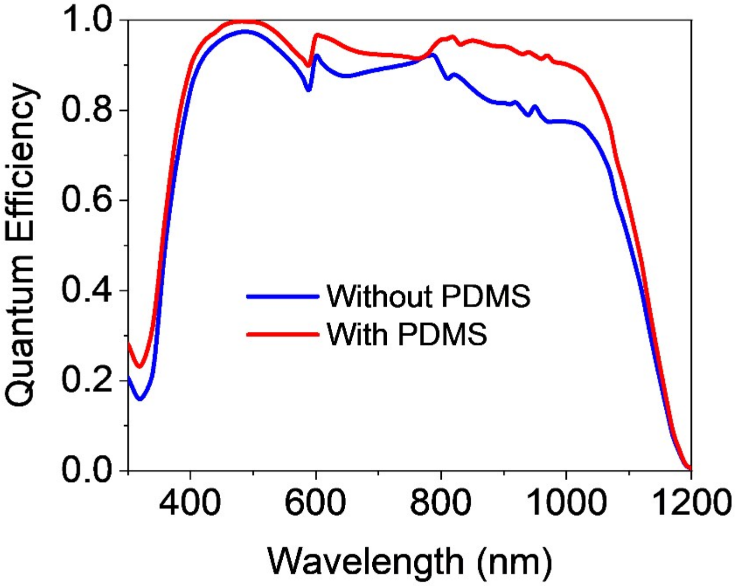

Fig. S4 A comparison of the calculated total quantum efficiency of perovskite/silicon tandem solar cell without and with PDMS optical incoupler.
